# Supplementary material for: A Humanized Yeast Phenomic Model of Deoxycytidine Kinase to Predict Genetic Buffering of Nucleoside Analog Cytotoxicity
Source: Genes (Basel). 2019 Sep 30;10(10):770. doi: 10.3390/genes10100770 (PMC6826991; doi:10.3390/genes10100770)
Supplement: Supplementary file 1 [file genes-10-00770-s001.zip › Additional_Files/Additional_File7_GOTermHeatmaps.zip!/Additional_File7_GOTermHeatmaps/hydrogen_ion_transmembrane_transporter_activity.pdf]

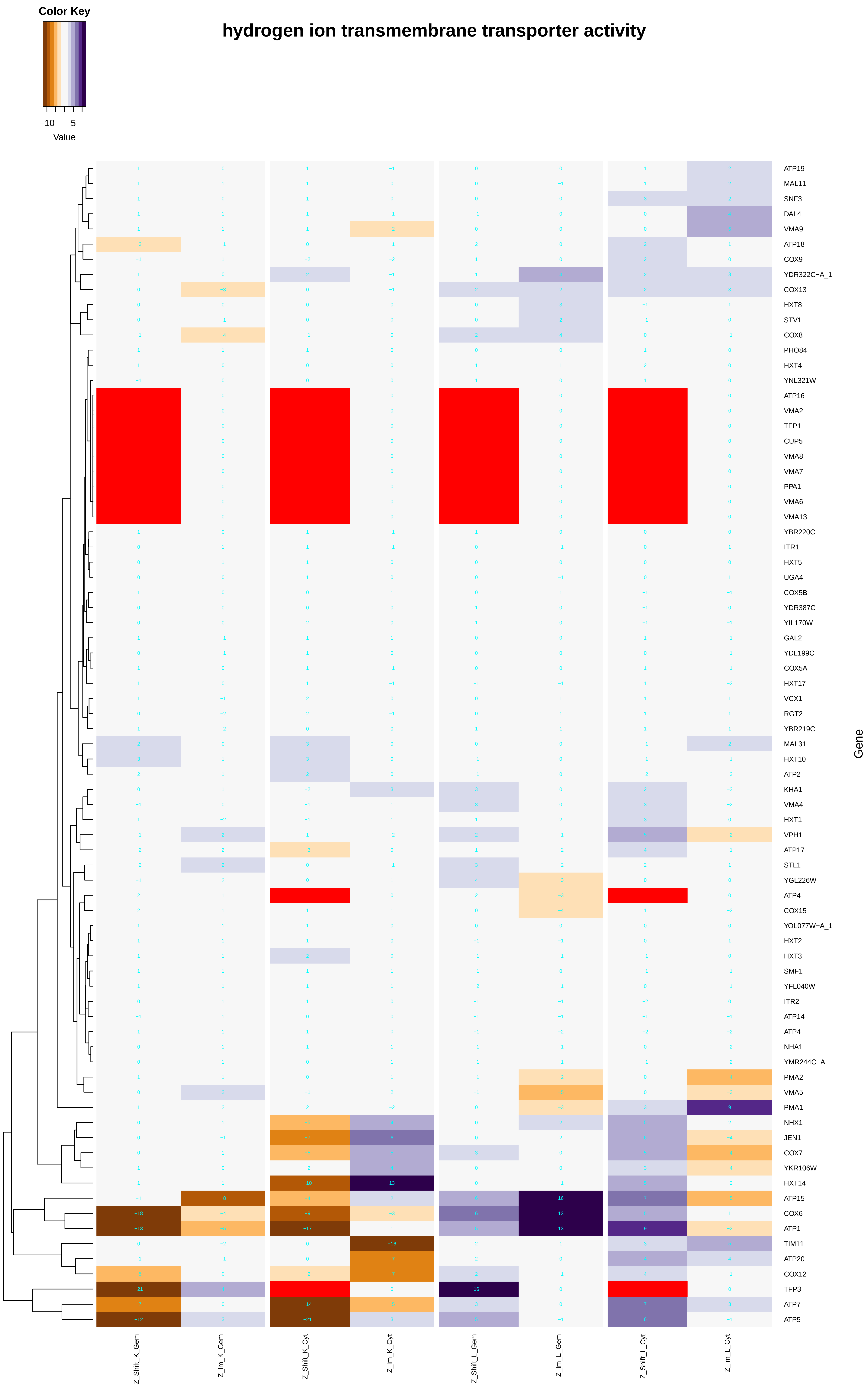
hydrogen ion transmembrane transporter activity
Color Key
−10
5
Value
ATP19
MAL11
SNF3
DAL4
VMA9
ATP18
COX9
YDR322C−A_1
COX13
HXT8
STV1
COX8
PHO84
HXT4
YNL321W
ATP16
VMA2
TFP1
CUP5
VMA8
VMA7
PPA1
VMA6
VMA13
YBR220C
ITR1
HXT5
UGA4
COX5B
YDR387C
YIL170W
GAL2
YDL199C
COX5A
HXT17
VCX1
RGT2
YBR219C
MAL31
HXT10
ATP2
KHA1
VMA4
HXT1
VPH1
ATP17
STL1
YGL226W
ATP4
COX15
YOL077W−A_1
HXT2
HXT3
SMF1
YFL040W
ITR2
ATP14
ATP4
NHA1
YMR244C−A
PMA2
VMA5
PMA1
NHX1
JEN1
COX7
YKR106W
HXT14
ATP15
COX6
ATP1
TIM11
ATP20
COX12
TFP3
ATP7
ATP5
Gene
Z_Shift_K_Gem
Z_Im_K_Gem
Z_Shift_K_Cyt
Z_Im_K_Cyt
Z_Shift_L_Gem
Z_Im_L_Gem
Z_Shift_L_Cyt
Z_Im_L_Cyt

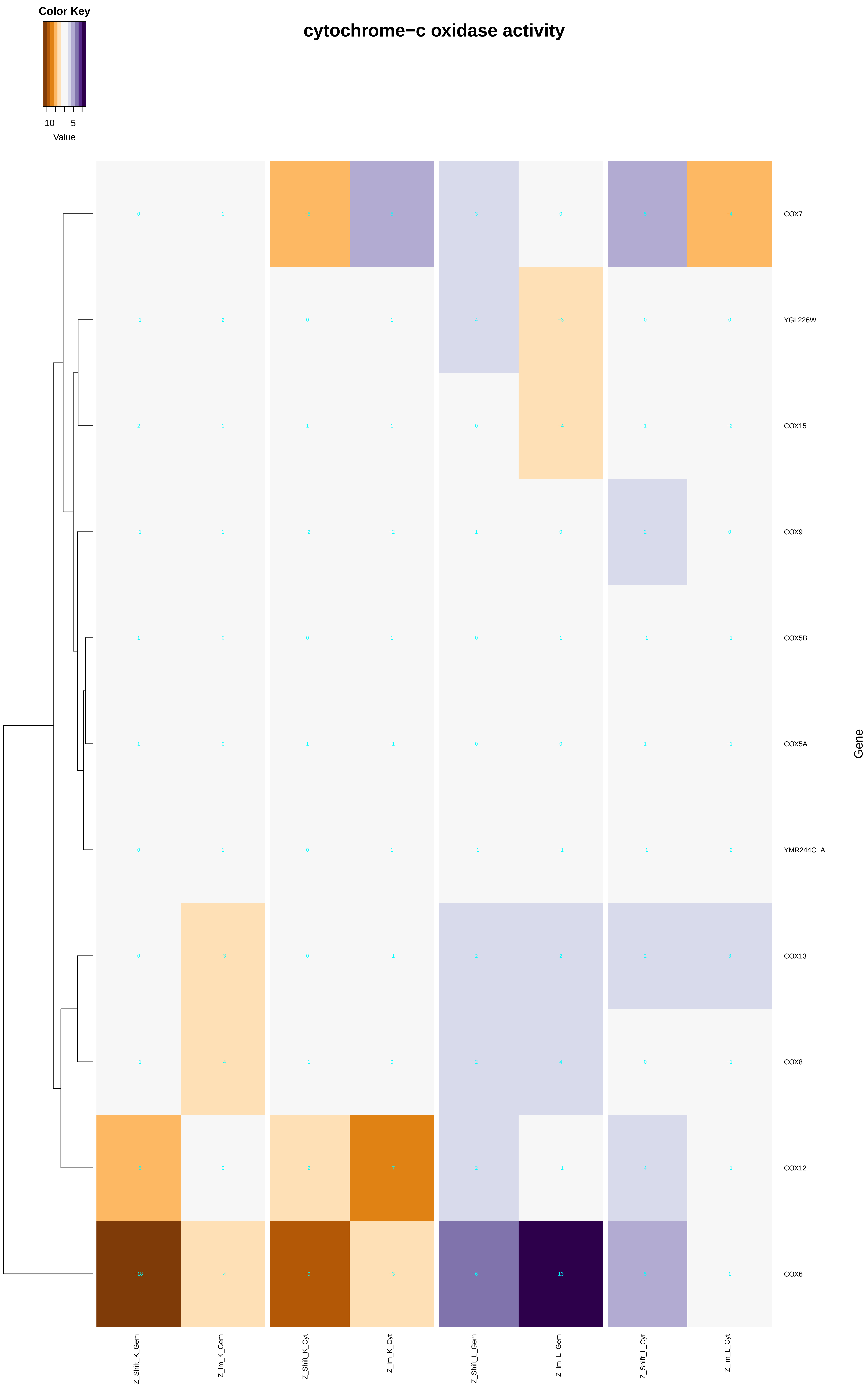
cytochrome−c oxidase activity
Color Key
−10
5
Value
Gene
COX7
YGL226W
COX15
COX9
COX5B
COX5A
YMR244C−A
COX13
COX8
COX12
COX6
Z_Shift_K_Gem
Z_Im_K_Gem
Z_Shift_K_Cyt
Z_Im_K_Cyt
Z_Shift_L_Gem
Z_Im_L_Gem
Z_Shift_L_Cyt
Z_Im_L_Cyt

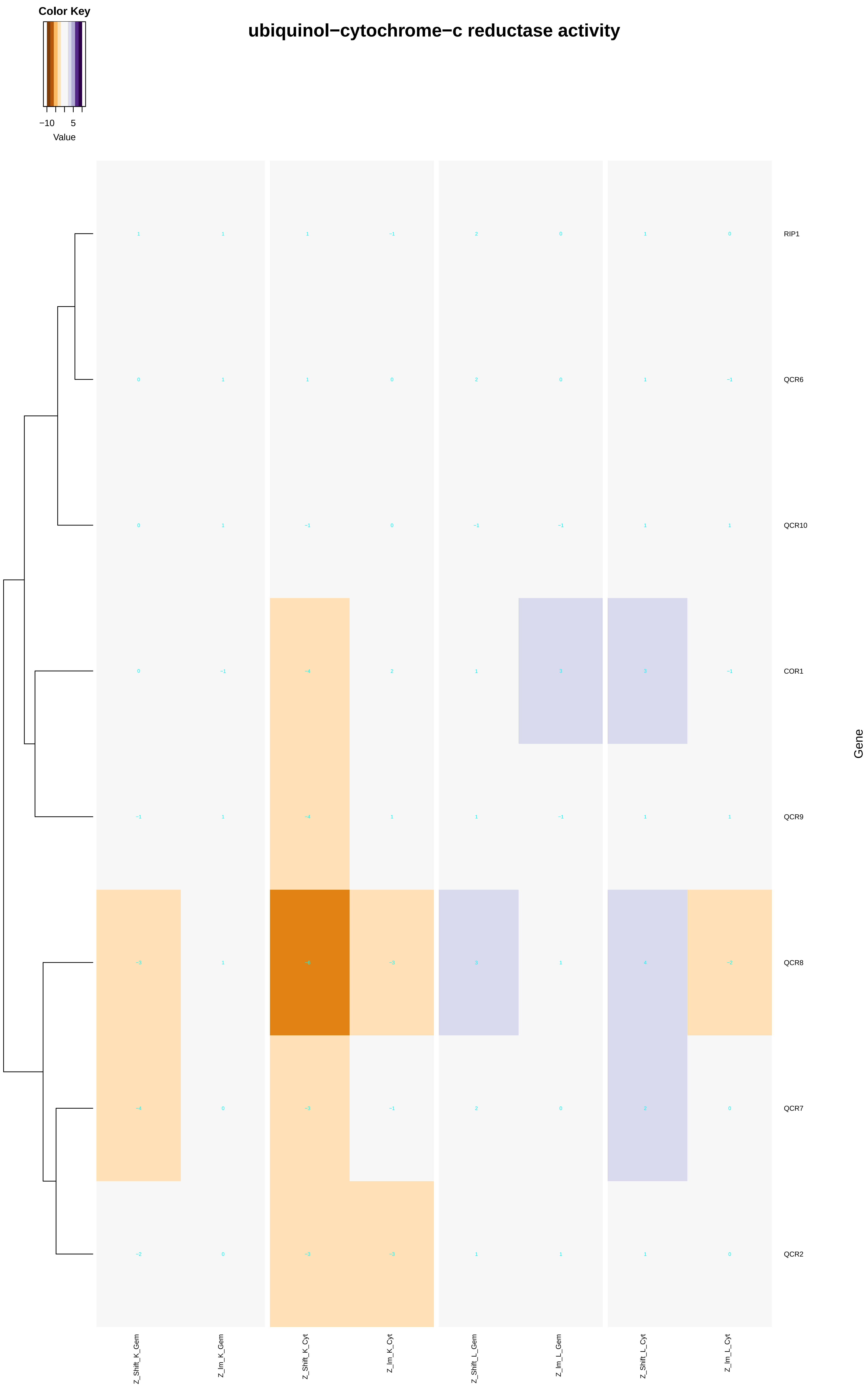
ubiquinol−cytochrome−c reductase activity
Color Key
−10
5
Value
RIP1
QCR6
QCR10
COR1
QCR9
QCR8
QCR7
QCR2
Gene
Z_Shift_K_Gem
Z_Im_K_Gem
Z_Shift_K_Cyt
Z_Im_K_Cyt
Z_Shift_L_Gem
Z_Im_L_Gem
Z_Shift_L_Cyt
Z_Im_L_Cyt

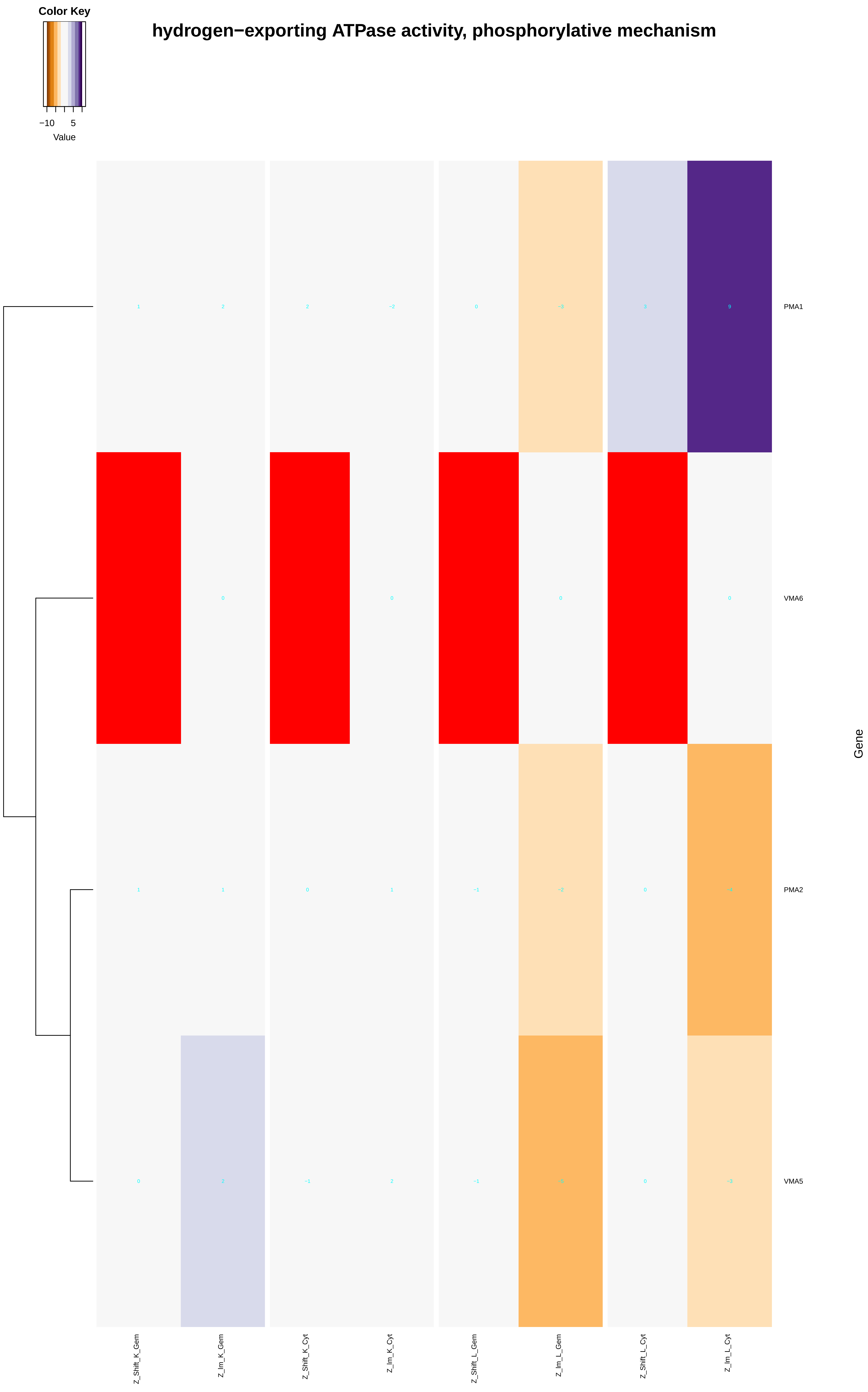

hydrogen−exporting ATPase activity, phosphorylative mechanism
Color Key
−10
5
Value
PMA1
VMA6
PMA2
VMA5
Gene
Z_Shift_K_Gem
Z_Im_K_Gem
Z_Shift_K_Cyt
Z_Im_K_Cyt
Z_Shift_L_Gem
Z_Im_L_Gem
Z_Shift_L_Cyt
Z_Im_L_Cyt

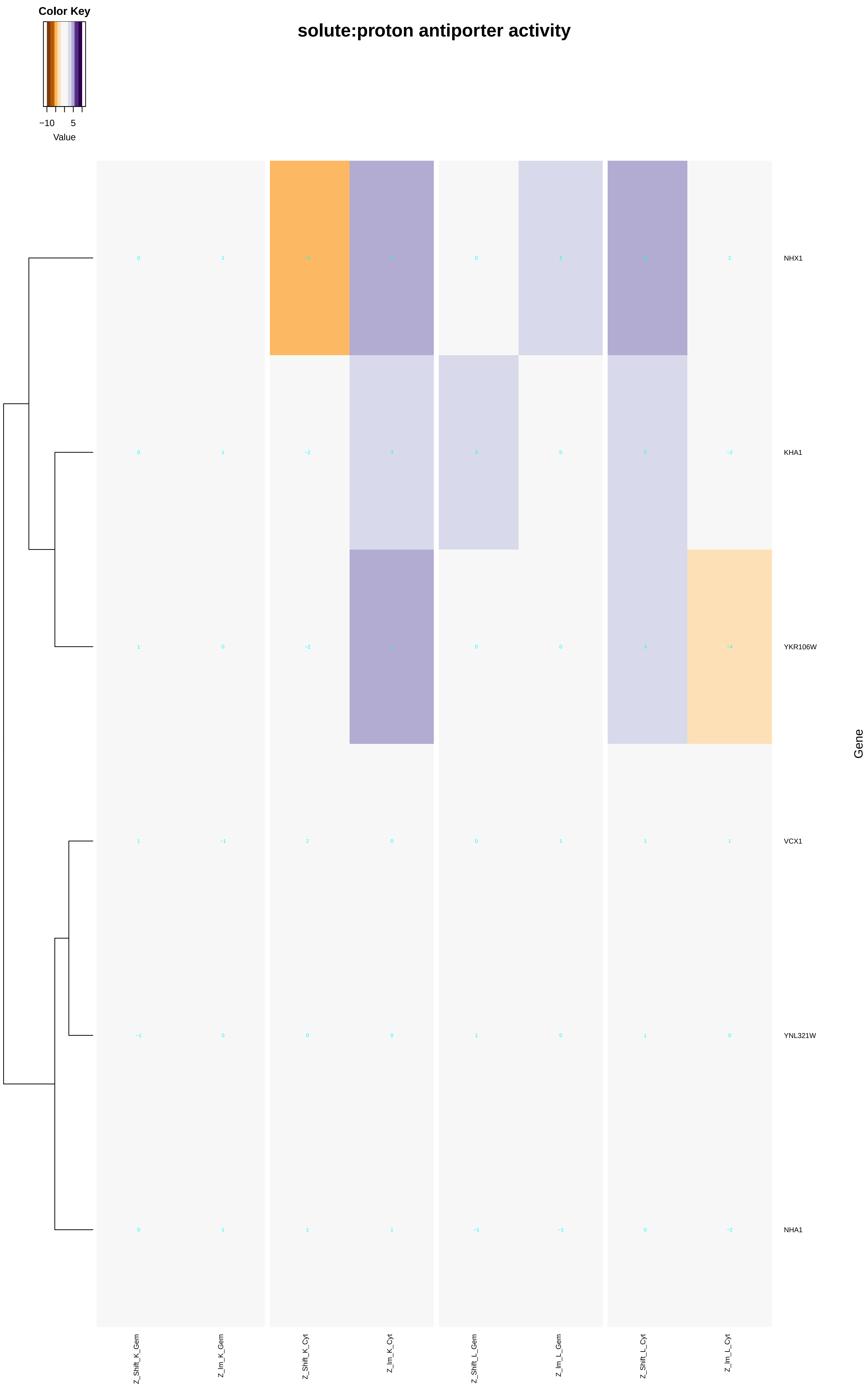

Color Key
−10
5
Value
solute:proton antiporter activity
NHX1
KHA1
YKR106W
VCX1
YNL321W
NHA1
Gene
Z_Shift_K_Gem
Z_Im_K_Gem
Z_Shift_K_Cyt
Z_Im_K_Cyt
Z_Shift_L_Gem
Z_Im_L_Gem
Z_Shift_L_Cyt
Z_Im_L_Cyt

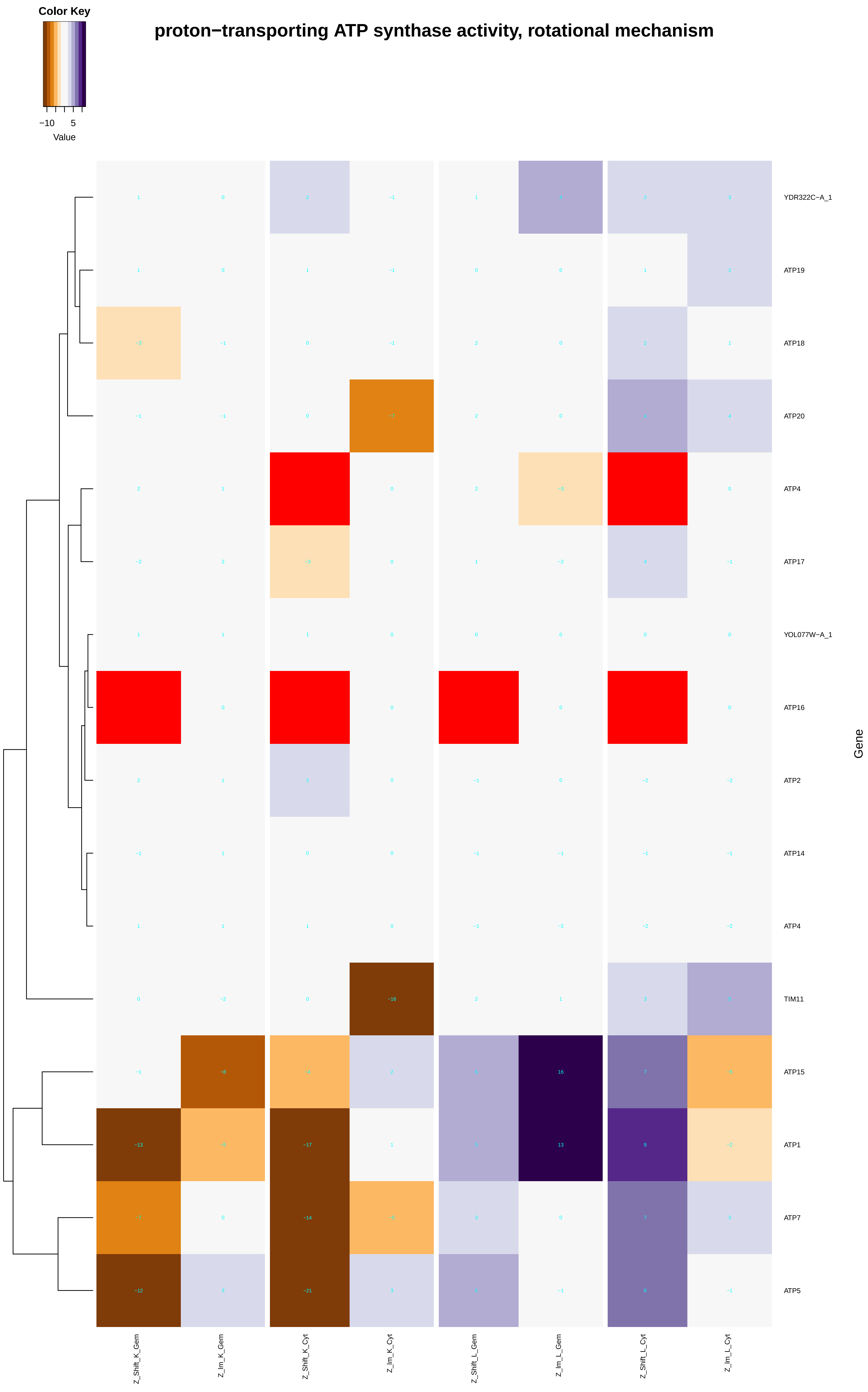
proton−transporting ATP synthase activity, rotational mechanism
Color Key
−10
5
Value
YDR322C−A_1
ATP19
ATP18
ATP20
ATP4
ATP17
YOL077W−A_1
ATP16
ATP2
ATP14
ATP4
TIM11
ATP15
ATP1
ATP7
ATP5
Gene
Z_Shift_K_Gem
Z_Im_K_Gem
Z_Shift_K_Cyt
Z_Im_K_Cyt
Z_Shift_L_Gem
Z_Im_L_Gem
Z_Shift_L_Cyt
Z_Im_L_Cyt

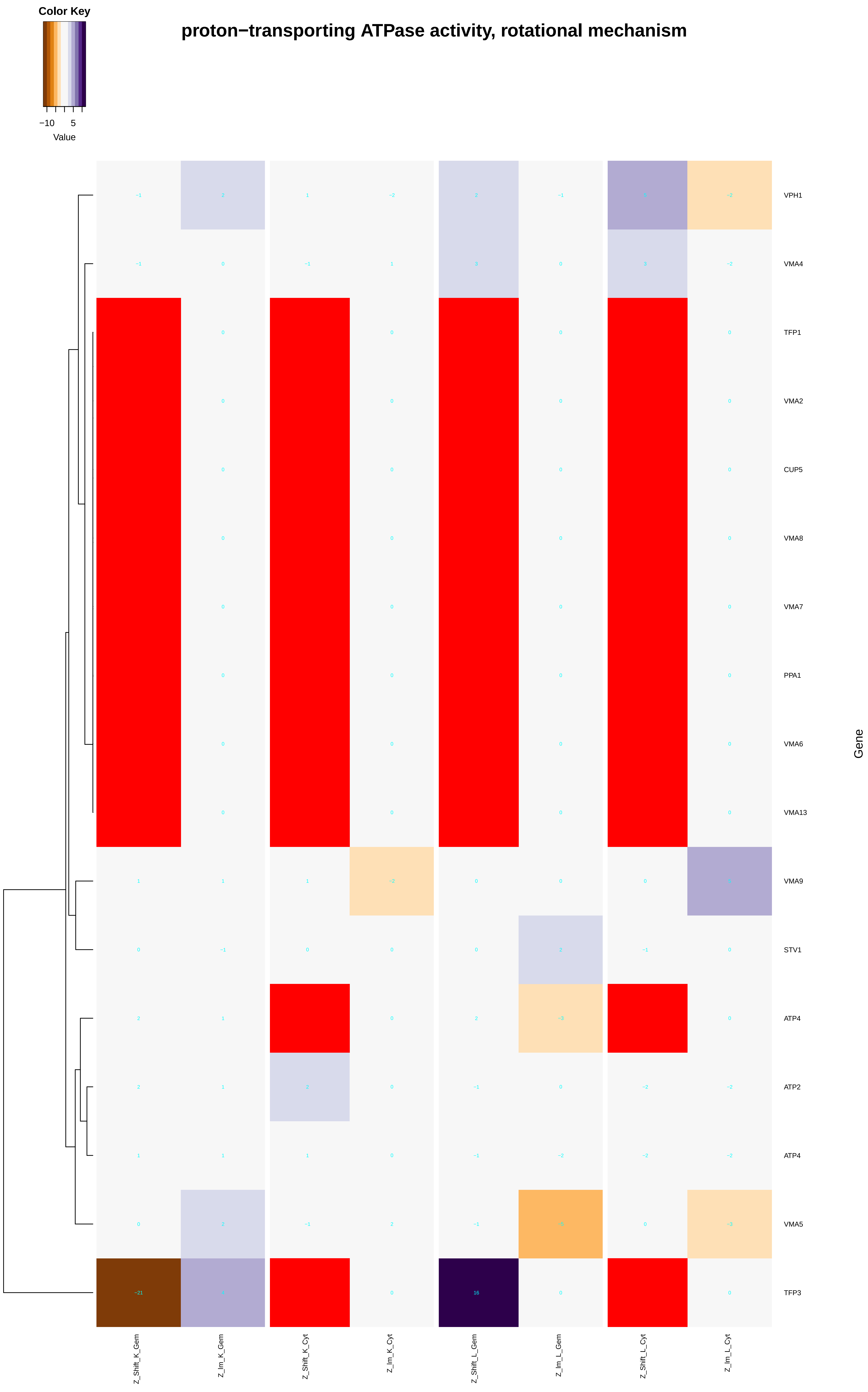
Color Key
−10
5
Value
proton−transporting ATPase activity, rotational mechanism
VPH1
VMA4
TFP1
VMA2
CUP5
VMA8
VMA7
PPA1
VMA6
VMA13
VMA9
STV1
ATP4
ATP2
ATP4
VMA5
TFP3
Gene
Z_Shift_K_Gem
Z_Im_K_Gem
Z_Shift_K_Cyt
Z_Im_K_Cyt
Z_Shift_L_Gem
Z_Im_L_Gem
Z_Shift_L_Cyt
Z_Im_L_Cyt

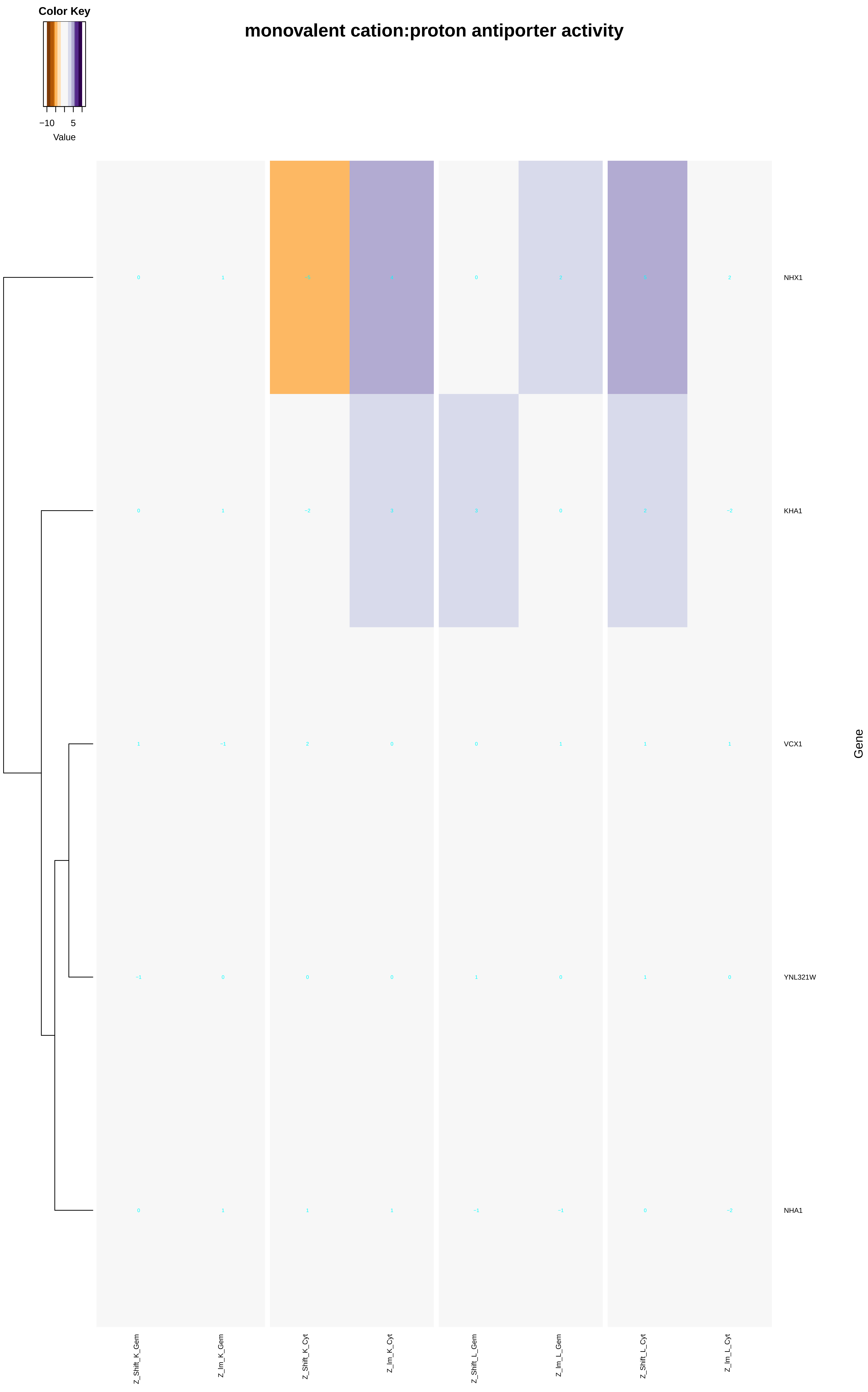

Color Key
−10
5
Value
monovalent cation:proton antiporter activity
NHX1
KHA1
VCX1
YNL321W
NHA1
Gene
Z_Shift_K_Gem
Z_Im_K_Gem
Z_Shift_K_Cyt
Z_Im_K_Cyt
Z_Shift_L_Gem
Z_Im_L_Gem
Z_Shift_L_Cyt
Z_Im_L_Cyt

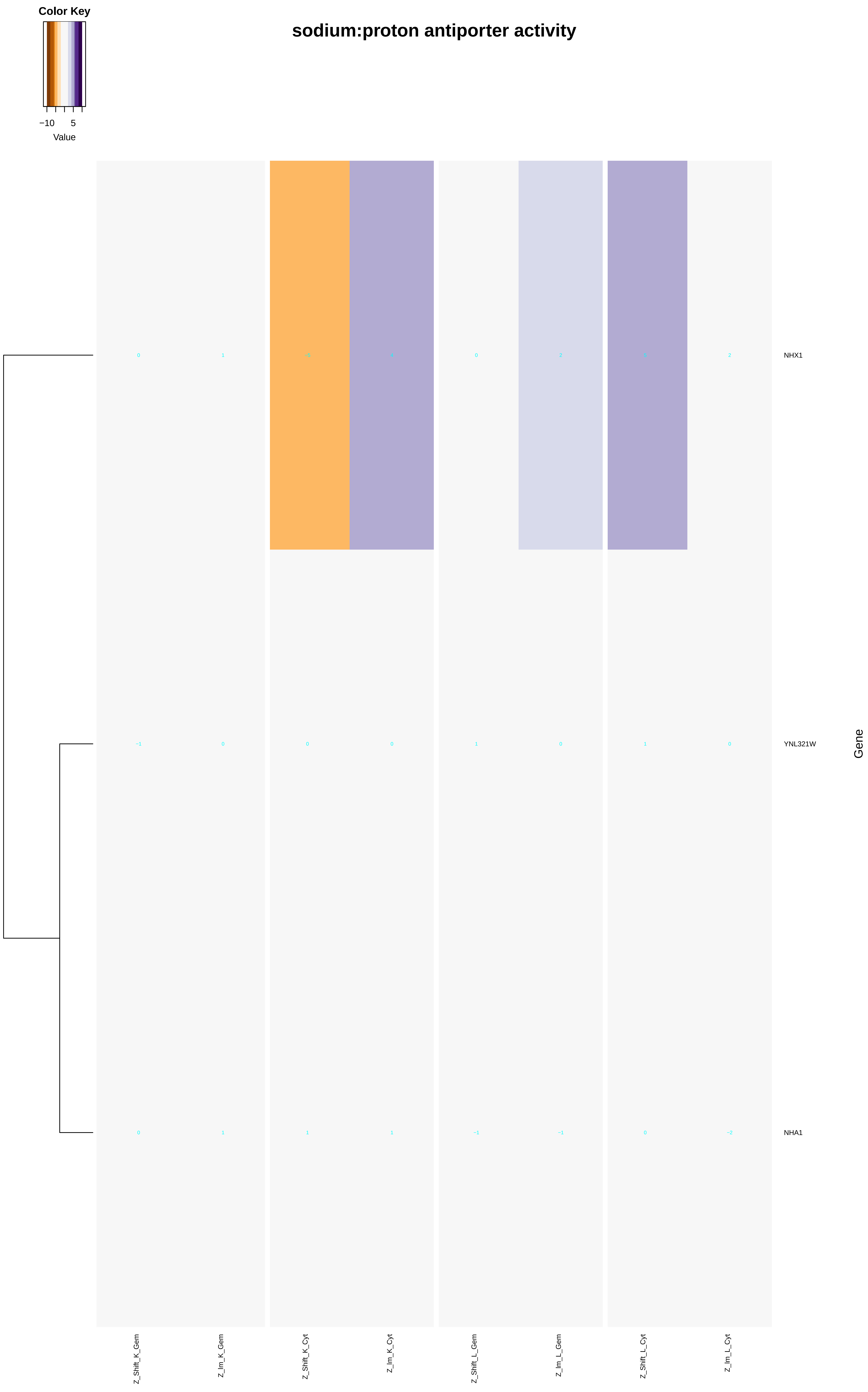
sodium:proton antiporter activity
Color Key
−10
5
Value
NHX1
YNL321W
NHA1
Gene
Z_Shift_K_Gem
Z_Im_K_Gem
Z_Shift_K_Cyt
Z_Im_K_Cyt
Z_Shift_L_Gem
Z_Im_L_Gem
Z_Shift_L_Cyt
Z_Im_L_Cyt

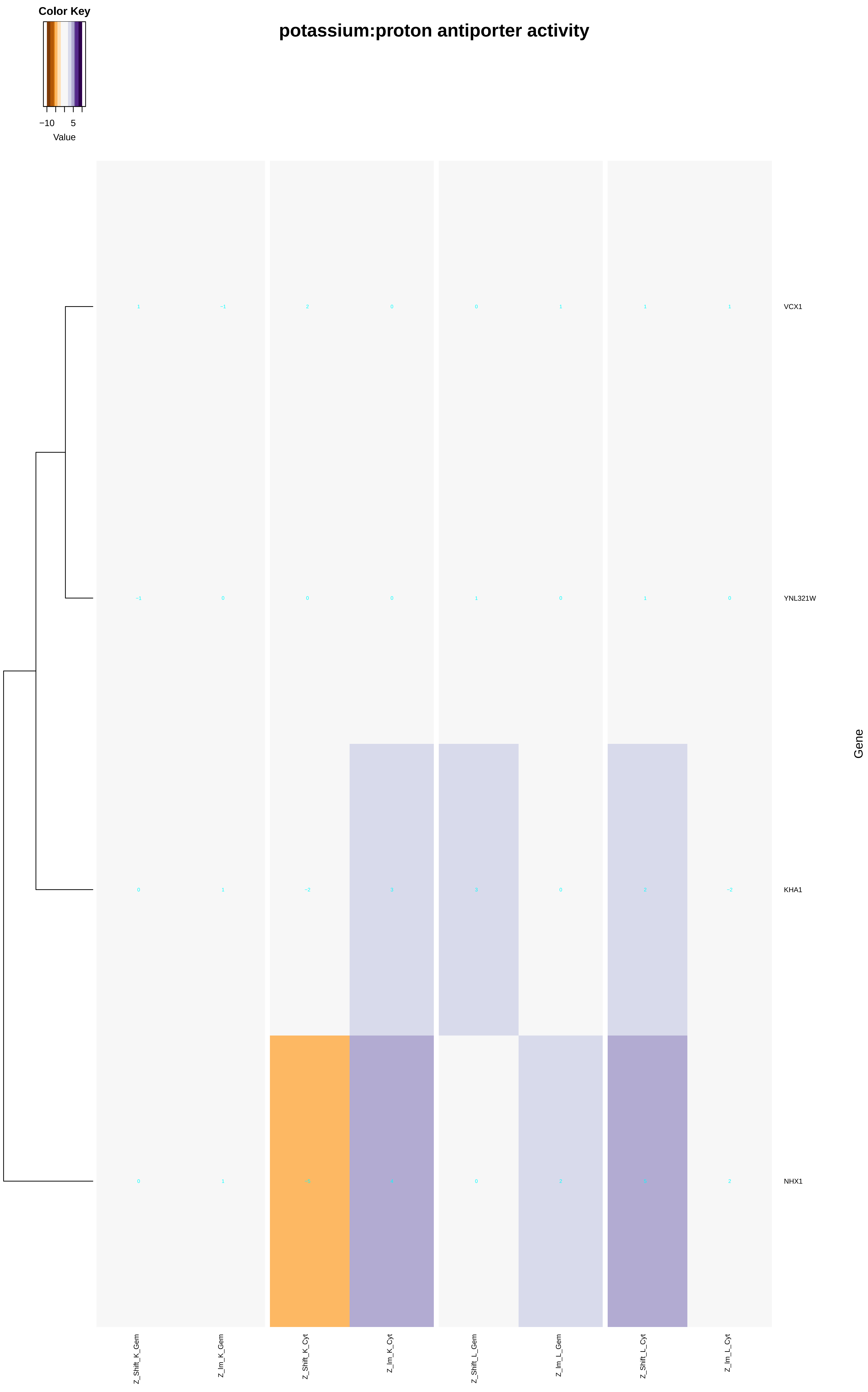
Color Key
−10
5
Value
potassium:proton antiporter activity
1
−1
2
0
0
1
1
1
VCX1
−1
0
0
0
1
0
1
0
YNL321W
0
1
−2
3
3
0
2
−2
KHA1
0
1
−5
4
0
2
5
2
NHX1
Gene
Z_Shift_K_Gem
Z_Im_K_Gem
Z_Shift_K_Cyt
Z_Im_K_Cyt
Z_Shift_L_Gem
Z_Im_L_Gem
Z_Shift_L_Cyt
Z_Im_L_Cyt
